# Sympatric Fagaceae Segregate Rodent Scatter-Hoarding Services for Similar Seed Dispersal Effectiveness in a Subtropical Forest in China

**DOI:** 10.3390/ani16193145

**Published:** 2026-10-08

**Authors:** Jifa Cui, Jie Chen

**Affiliations:** 1 School of Environment & Resource, Xichang University, Xichang 615000, China; 2 School of Resources and Environmental Engineering, Anhui University, Hefei 230601, China

**Keywords:** coexistence, Fagaceae family, niche differentiation, plant–rodent interactions, seed germination, seedling survival, seed fate

## Abstract

In forest ecosystems where seed dispersal is predominantly mediated by scatter-hoarding rodents, sympatric plants compete for these limited dispersal services. This competition can drive niche differentiation, prompting the question of how ecologically similar species achieve coexistence. To address this, we conducted a seed provisioning experiment in the Ailao Mountains, Yunnan Province, China. We released 4500 tagged seeds from three congeneric species of stone oaks (*Lithocarpus hancei*, *Lithocarpus xylocarpus*, and *Lithocarpus pachyphyllus*; Fagaceae) to compare their seed dispersal effectiveness (SDE) and identify the divergent strategies that may facilitate their coexistence. Seeds of all three species were more likely to be removed than eaten in situ. Among the removed seeds, the proportion of cached versus eaten did not differ significantly among species. *L. pachyphyllus* exhibited both a higher seed removal rate and greater dispersal distances compared to *L. xylocarpus* and *L. hancei*. However, in contrast to its congeners, *L. pachyphyllus* seeds were more frequently dispersed to shrub microsites rather than open areas. Despite these contrasting dispersal patterns, the three species ultimately achieved statistically similar SDE. These results demonstrate that congeneric species can exploit rodent dispersal services in distinct ways while attaining equivalent dispersal effectiveness. This niche segregation in dispersal strategy represents a potential form of niche partitioning that could theoretically promote coexistence among ecologically similar species.

## Simple Summary

In forests where scatter-hoarding rodents dominate seed dispersal, sympatric plants compete for limited dispersal services, raising the question of how ecologically similar congeners coexist. We conducted a tagged seed experiment with 4500 seeds from three sympatric stone oak species (*Lithocarpus hancei*, *Lithocarpus xylocarpus*, *Lithocarpus pachyphyllus*) in China’s Ailao Mountains, comparing their seed dispersal effectiveness (SDE) and coexistence strategies. All species had higher seed removal than in situ consumption, with no interspecific differences in the cached-to-eaten ratio of removed seeds. *L. pachyphyllus* showed higher seed removal rates and longer dispersal distances, and its seeds preferentially dispersed to shrub microsites, whereas the other two species favored open areas. Despite divergent dispersal strategies, the three species achieved equivalent SDE. Our results indicate that congeneric plants can realize niche segregation via distinct rodent-mediated dispersal patterns, facilitating the coexistence of similar species.

## 1. Introduction

An organism’s basic requirements define its ‘fundamental niche’, which Hutchinson termed an ‘n-dimensional hypervolume’ [1,2]. Within this niche space, each individual organism must compete with conspecifics and other species with similar requirements [3]. Consequently, individuals or species with the best fitting traits will achieve a selective advantage by either winning contests directly or by depleting the limiting shared resource more efficiently than its competitor [4]. This results in ‘competitive exclusion’, where no two species can occupy exactly the same niche hypervolume (Gause’s law; [5]). Instead, species adopt subtly different alternate life-history strategies, partitioning contested resources so that each occupies a unique ‘realized niche’ [6]. In turn, tactical phenotypic plasticity can reduce intra-specific conflict [7]. Thanks to phenotypic plasticity, organisms from different taxonomic groups can adapt to successfully coexist [8,9]. The diversity of taxa with discrete differentiated niches within an ecosystem thus represents a type of evolutionary ‘bet-hedging’ [10].

Recent research has revealed that plants segregate their essential and fundamentally similar resource requirements along various environmental niche axes (e.g., gradients of light, soil moisture and root depth, and the partitioning of soil nutrients, etc.), restricting the realized niche of some species to less than half of their fundamental niche [11]. Nevertheless, the role of competition for zoochorous services in driving the tactical partitioning of seed dispersal traits between similar plant species has not been considered. This is important with regard to forest regeneration and for the conservation of the ecological communities that reside in these important habitats [12,13].

Seed dispersal effectiveness (SDE) provides a mechanistic framework to evaluate the evolutionary and ecological characteristics of zoochorous seed dispersal. SDE synthesizes the quantity and quality of animal-mediated dispersal each plant species receives [14,15]. Ultimately, each plant species in a community will seek to achieve the greatest share of zoochorous seed dispersal that it can, matching or exceeding its niche competitors. While the drivers of SDE have received attention from the perspective of the animal agents involved [15,16], the differentiation of SDE among interacting sympatric plant species competing for the services of animals remains to be fully resolved, especially in relation to mutualisms with scatter-hoarding rodents.

Scatter-hoarding rodents play a key role in the processes of seed dispersal and successful seed germination [17,18]. Rodents harvest seeds either by consuming them immediately as a source of nutrition or by carrying seeds to more distant locations to scatter-hoard them as a food cache intended for later consumption, but often forgotten or un-utilized [18]. While seed predation can destroy seed viability [19], by placing seeds at local microsites that generally favor seedling establishment [20], rodent scatter-hoarding behavior can have a profoundly beneficial influence on seedling establishment, plant community construction, and forest regeneration [21,22].

Plants compete for rodent seed scatter-hoarding services through the evolution of seed traits that appeal to rodents in different ways [23,24]. This results in differences in both the quantity of seeds dispersed and the quality of this dispersal in terms of the percentage of seeds that successfully produce “seedlings” [14,25]. For example, smaller seeds are more likely to be eaten in situ while larger seeds are more likely to be transported, cached, and dispersed over a longer distance [18,26]. A high tannin content also resists seed predation [27,28].

However, the differentiation of SDE among interacting sympatric plant species competing for the services of animals remains to be fully resolved, especially in relation to mutualisms with scatter-hoarding rodents. The overall objective of this study was to explore whether sympatric congeneric tree species can achieve similar seed dispersal effectiveness through dispersal-niche partitioning when competing for limited rodent scatter-hoarding services. In this study, we compared the differences in key components of rodent-mediated seed dispersal and the final seed dispersal effectiveness (SDE) among three congeneric species of stone oaks (*Lithocarpus hancei*, *Lithocarpus xylocarpus*, and *Lithocarpus pachyphyllus*; Fagaceae) that share similar phylogeny and dispersal syndromes. In our subtropical evergreen broad-leaved forest field site in the Ailao Mountains, Yunnan Province, the fruiting periods of these species overlap substantially, heightening the intensity of competition for limited rodent-mediated seed dispersal services. Based on this system, we tested two hypotheses: (1) that the dimensions of dispersal (i.e., harvest rate, caching rate, dispersal distance, and caching microsite) would differ among these three *Lithocarpus* spp.; but nevertheless, (2) despite these differences in dispersal processes, all three species would ultimately achieve statistically similar overall SDE. The latter hypothesis is grounded in the concept of dispersal niche partitioning, wherein sympatric species may achieve comparable fitness outcomes via divergent dispersal pathways, thereby representing a potential mechanism that could contribute to species coexistence. However, we acknowledge that direct demographic evidence would be required to confirm this mechanism.

## 2. Materials and Methods

### 2.1. Study Site and Species

This study was conducted in a subtropical evergreen broadleaf forest in the Ailao Mountains, southwestern China (24°32′ N, 101°01′ E, elevation 2045 m) from November 2020 until May 2021. Annual temperature averages 11.7 °C, with an average annual precipitation of 1923 mm, much of which falls in the rainy season from May to October. The forest is dominated by trees of the families Fagaceae, Lauraceae, Theaceae, and Magnoliaceae.

The three focal *Lithocarpus* species, i.e., *L. hancei*, *L. xylocarpus*, and *L. pachyphyllus*, co-occur in this forest and exhibit partially overlapping fruiting periods. *L. xylocarpus* and *L. hancei* are dominant canopy species, whereas *L. pachyphyllus* is a relatively uncommon sub-canopy tree. Their seeds are primarily dispersed by scatter-hoarding rodents [26,27,28,29,30,31]. In this area, the dominant rodent species responsible for seed removal and dispersal are the Chinese white-bellied rat (*Niviventer confucianus* (Milne-Edwards, 1871)) [32], the South China field mouse (*Apodemus draco* (Barrett-Hamilton, 1900) [33], and the large white-bellied rat (*Niviventer excelsior* (Thomas, 1911)) [34], among others [26,30,31,35,36]. Although other mammals such as deer (*Cervus* spp.) and wild boar (*Sus scrofa* (Linnaeus, 1758)) [37] occur in the region and may occasionally consume *Lithocarpus* fruits, their foraging leaves distinct, non-scatter-hoarding signs (e.g., trampling or rooting). Note that our seed-tracking data cannot attribute seed-dispersal events to individual rodent species; we treat the whole local rodent assemblage as a functional “black box”.

For our experiments, we collected naturally mature *L. xylocarpus*, *L. hancei*, and *L. pachyphyllus* seeds from the forest floor in the research area in November 2020. We then selected intact seeds using water floatation combined with visual inspection [38,39]. Buoyant seeds were assumed to be empty (and thus devoid of any nutritional value) and discarded. Seed mass (*n* = 1500 per species) was greatest for *L. xylocarpus* (mean ± SD; 4.36 ± 1.77 g), followed by *L. pachyphyllus* (2.52 ± 0.57 g) and *L. hancei* (1.54 ± 0.38 g). Seeds were individually marked for single-seed identification: a 0.6-mm-diameter hole was drilled at the base of each seed. Each seed was tagged to a uniquely coded white plastic tag (2.5 × 3.5 cm) with a thin steel thread (0.2 mm in diameter, 15 cm in length) [27,30,40]. Each tag bore a distinct unique identification number, which allowed us to unambiguously recognise each individual seed during field surveys and prevented repeated counting of the same seed dispersal event. Previous research has shown that these tags tend not to affect rodent seed handling behavior [41].

### 2.2. Seed Release and Tracking

In December 2020, 15 trees of each tree species were selected, spaced > 30 m apart. This spacing exceeds the typical seed transport distance of local rodents (≤20 m, based on prior studies in this forest), thus ensuring independence among experimental units [31,35,36,42]. One seed release point was randomly set below the canopy of each selected tree. At each point, 100 seeds of the same species as the focal tree were placed, arranged equidistantly along the perimeter of a 30 cm diameter circle. A sample size of 100 seeds per release point provides sufficient statistical power to quantify seed-fate proportions, while the circular layout minimizes directional bias in rodent seed access. In total, 4500 seeds were released (100 seeds × 15 trees × 3 species). Seed tracking surveys were performed only once at the end of the overwintering period; no intermediate repeated searches were conducted during the winter. In May 2021, we surveyed the overwintering fate of each seed by conducting intensive and comprehensive searches within a 30 m radius of each release point tree. This 30-m search radius covers the maximum reported dispersal distance for local rodent dispersers in this forest. In addition, we conducted random searches outside of this 30 m radius to find as many more extensively transported seeds as possible, to recover seeds dispersed beyond the regular search radius, and reduce missing-data bias [31].

Seed fate was categorized into harvested and ignored (i.e., seeds remaining intact at their initial release point). Harvested seeds were then divided into eaten in situ or removed (translocated) away from their original seed releasing point. Removed seeds included those cached (i.e., seeds either placed on the ground surface or cached lightly buried in soil), those eaten after being removed, and missing seeds (i.e., seeds that were not found with unknown fate). For those removed seeds that were found, the distance between the seed release point to the relocation point was measured linearly using a laser rangefinder. A few (<0.8%) harvested seeds were found buried in soil in situ, and these seeds were also classified as seeds cached, with their dispersal distance recorded as “0 m”. We also investigated the microhabitat types into which each harvested seed was removed. We followed the habitat categories used in a previous study [43]: (i) open, (ii) within 10 cm of the canopy of a shrub, and (iii) placed directly under the canopy of a shrub; however, the latter two shrub categories were combined for analysis because <1.69% of seeds were removed to under a shrub.

### 2.3. Data Analyses

We defined each response index as follows: harvest rate = number of harvested seeds/total released seeds; removal rate = number of removed seeds/total harvested seeds; caching rate = number of cached seeds/total found removed seeds. Dispersal distance referred to the linear Euclidean distance measured from each seed-release point to the final relocated position of individual seeds. The microhabitat-use index represented the proportion of removed seeds that were transported to open versus shrub microhabitats.

We calculated the seed dispersal effectiveness (SDE) following the method described in previous studies [14,25]: SDE = $\frac{\mathrm{Number of harvested seeds}}{\mathrm{Total number of released seeds}}$ × $\frac{\mathrm{Number of removed seeds}}{\mathrm{Number of harvested seeds}}$ × $\frac{\mathrm{Number of cached seeds}}{\mathrm{Number of removal seeds that were found}}$.

To analyze differences among species, a series of generalized linear mixed-effects models (GLMMs) with Tukey’s HSD multiple comparisons were constructed with ‘species’ as the independent variable, various seed fates-related indices as response variables, and ‘seed release point’ as a random effect for all indices except SDE. The following six indexes were tested: (1) harvest rate; (2) removal rate; (3) caching rate; (4) dispersal distance; (5) seed dispersal effectiveness (SDE); and (6) percentage of seeds removed into open areas versus shrubs. For indices (1)–(4) and (6), a binomial distribution was adopted, with a Gaussian distribution applied only for dispersal distance. For SDE, a generalized linear model (GLM) without the random effect was used instead of a GLMM, with a Gaussian distribution assumed. All analyses were performed in R 4.0.5 [44] using the ‘lme4’ and ‘multcomp’ packages.

## 3. Results

### 3.1. Fates of Dispersed Lithocarpus spp. Seed

**Figure 1:**
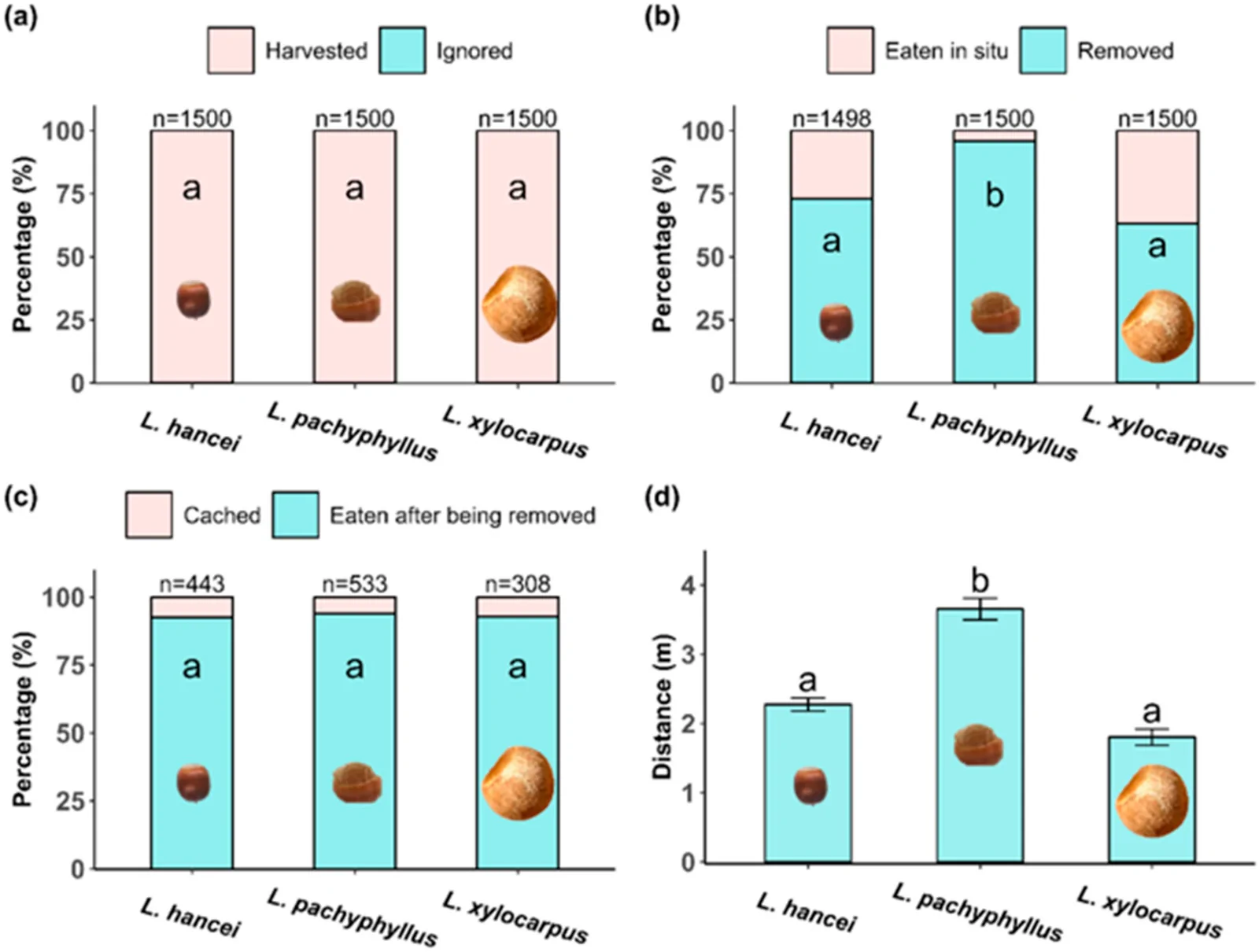
Seed removal and caching fates of the three *Lithocarpus* species. (**a**) seed harvested vs. ignored; (**b**) removed vs. eaten in situ; (**c**) cached vs. eaten after being removed; (**d**) dispersal distance (mean ± SE). Different lowercase letters indicate significant differences among species within each panel (*p* < 0.05).

There was no significant difference in the percentage of seed harvested (99.96%) vs. ignored (0.04%) among species (*P*_1_ = 1, *P*_2_ = 1, *P*_3_ = 1; Table S1, Figure 1a). Harvested seeds of all three *Lithocarpus* spp. were more likely to be removed than eaten in situ. In relation to our first hypothesis, this removal likelihood was significantly greatest for *L. pachyphyllus* (95.87% vs. 4.13%, *n* = 1500) compared to both *L. hancei* (72.96% vs. 27.04%, *n* = 1498) and *L. xylocarpus* (63.13% vs. 36.87%, *n* = 1500) (*P*_1_ < 0.001, *P*_2_ < 0.001), which were not significantly different from each other (*P*_3_ = 0.821; Table S1, Figure 1b). Of the harvested seeds that were removed, there was no significant difference in the percentage of seeds being cached vs. those eaten among species (*L. pachyphyllus* 6.00% vs. 94.00%, *n* = 533; *L. hancei* 7.45% vs. 92.55%, *n* = 443; and *L. xylocarpus* 7.14% vs. 92.86%, *n* = 308) (*P*_1_ = 0.933, *P*_2_ = 0.927, *P*_3_ = 0.754; Table S1, Figure 1c). The dispersal distance of *L. pachyphyllus* seed (3.65 ± 0.16 m, mean ± SE, *n* = 533) was significantly greater than for *L. hancei* (2.27 ± 0.09 m, *n* = 443) or *L. xylocarpus* (1.80 ± 0.12 m, *n* = 308) (*P*_1_ = 0.005, *P*_2_ < 0.001; Table S1, Figure 1d).

**Figure 2:**
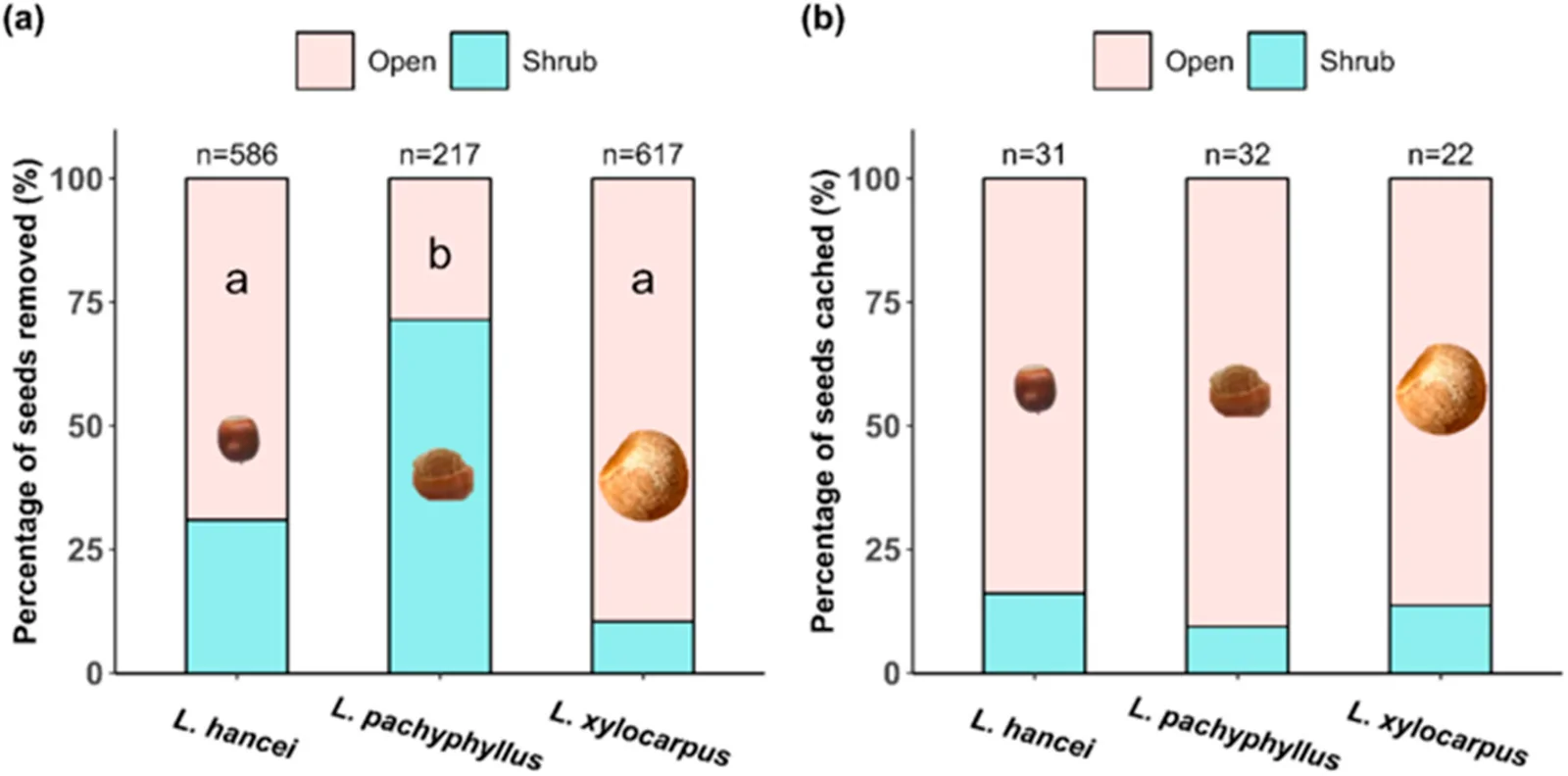
Microhabitat distribution of removed and cached seeds. (**a**) Proportion of removed seeds transported to shrub versus open microhabitats; (**b**) proportion of cached seeds cached in each of two microhabitat types—no statistical comparisons were performed due to small sample sizes. Different lowercase letters indicate significant differences among species within each panel (*p* < 0.05).

The relative percentage of seeds removed to shrubs versus open microhabitats also differed significantly among species (Table S1, Figure 2a). The likelihood of *L. pachyphyllus* seeds being removed to shrubs rather than open microhabitat (71.4% vs. 28.6%, *n* = 217) was greater than for either *L. hancei* (30.9% vs. 69.1%, *n* = 586) or *L. xylocarpus* (10.4% vs. 89.6%, *n* = 617) (*P*_1_ = 0.005, *P*_2_ < 0.001; Table S1, Figure 2a).

### 3.2. Seed Dispersal Effectiveness Similarity Between Lithocarpus spp.

**Figure 3:**
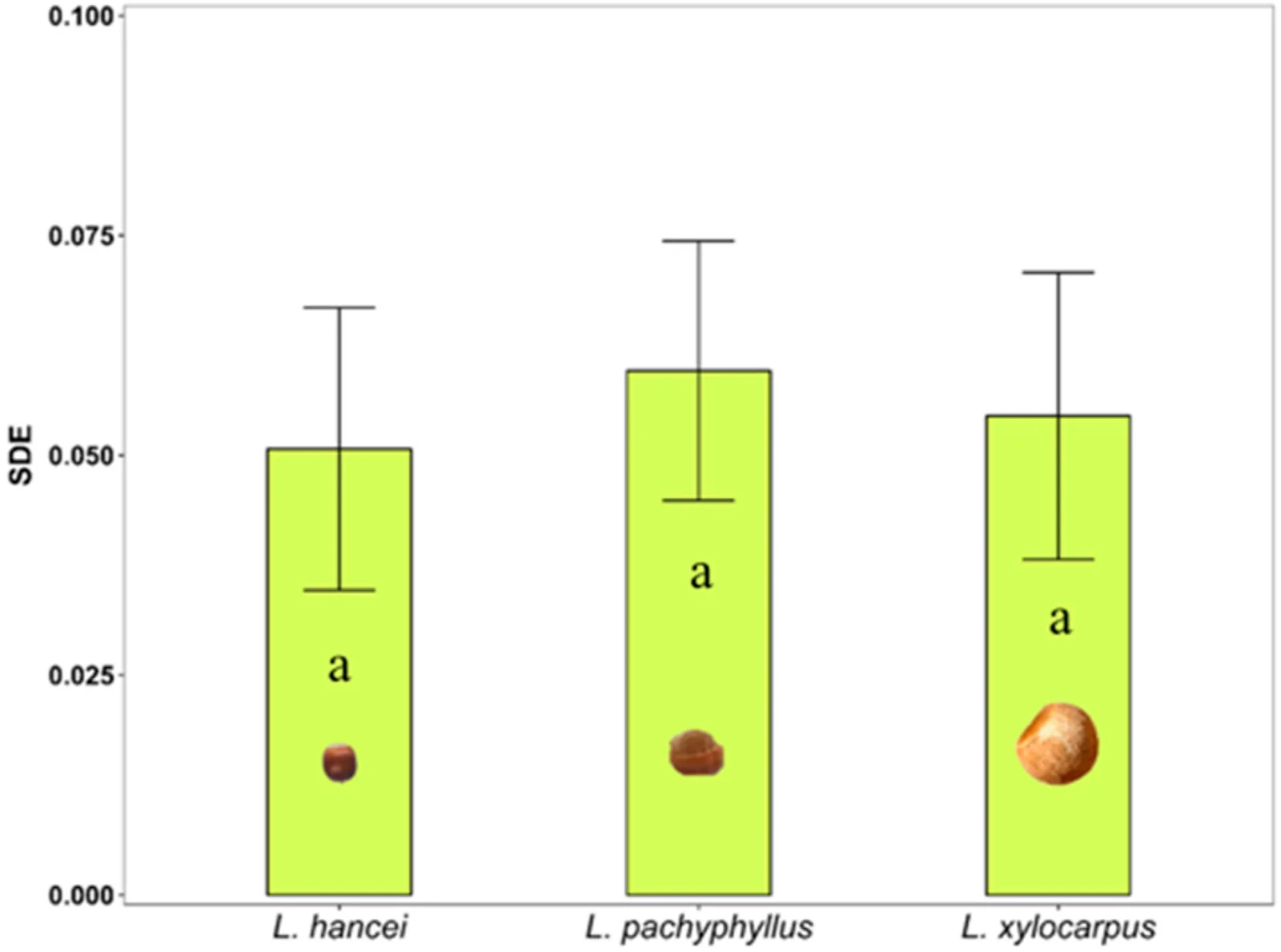
Seed dispersal effectiveness (SDE) of the three *Lithocarpus* species. Data are presented as mean ± SE. Different lowercase letters indicate significant differences among species within each panel (*p* < 0.05).

In support of our second hypothesis, seed dispersal effectiveness (SDE) did not differ significantly across the three study species (all *p* > 0.05; Table S1, Figure 3). Mean SDE values were as follows: *L. pachyphyllus* (mean ± SE: 0.060 ± 0.015; range 0–0.167), *L. xylocarpus* (0.054 ± 0.016; range 0–0.237), and *L. hancei* (0.051 ± 0.016; range 0–0.183). Pairwise comparisons further confirmed the absence of interspecific differences in SDE (*P*_1_ = 0.916, *P*_2_ = 0.984, *P*_3_ = 0.971; Table S1). Notably, this comparable overall SDE was observed despite marked inter-specific divergence in multiple dispersal-related variables documented in Section 3.1, including seed removal rates, dispersal distances, and microhabitat destinations of the removed seeds.

## 4. Discussion

In accord with our first hypothesis, all three congeneric species of *Lithocarpus* in our study utilized rodent dispersal services in distinct ways, indicating heterogeneity within the scatter-hoarding processes. Crucially, however, all three species achieved statistically similar levels of seed dispersal effectiveness (SDE). This pattern suggests that within this community, they partition the available rodent dispersal niche through divergent strategies, thereby supporting our second hypothesis.

To survive, plants must disperse seeds. Among allochorous species, this function is predominantly achieved either by reliance on the wind to disperse light weight seed (anemochory; with hydrochory among aquatic plant species) or reliance on mutualistic interactions with animals to provide seed dispersal [45]. Zoochorous seed dispersal can be considered a shared and potentially limiting resource among plants, resulting in exploitation competition among plant species relying on the services of the community of animals available to them. Therefore, how large numbers of diverse, competing plant species manage to coexist in an ecosystem remains a major unresolved question in community ecology [46].

Plant species-specific differences in seed traits result in differentials in seed dispersal distances and caching rates [47]. Furthermore, there can be considerable intraspecific heterogeneity in seed traits that also vary inter-annually [48]. This variability forms a potential basis for divergent dispersal strategies among species. One hypothesized mechanism for the coexistence of sympatric species relying on the same disperser guild is that trade-offs may occur across different stages of the dispersal process. For example, a species with a lower seed removal rate might compensate through higher caching rates or greater dispersal distances in a subset of its seeds [49], potentially leading to comparable net dispersal outcomes under specific conditions. The integrated outcome of these processes, i.e., the quality of seed dispersal, is ultimately reflected in seed fate and quantified as SDE [14]. Our study directly tested this compensatory hypothesis within a controlled experimental framework and found that despite contrasting dispersal pathways, the three congeneric *Lithocarpus* species achieved statistically similar SDE, providing a clear empirical case of such a trade-off system in action.

Interspecific differences in species’ seed traits are generally relatively stable, causing the seed selection behavior of rodents to also be broadly consistent [50,51]. Various studies have reported differences in rodent-mediated dispersal for sympatric tree species [52,53]. For instance, a previous study [52] found that three similar sympatric oak species (*Quercus aliena*, *Q. glandulifera*, and *Cyclobalanopsis engleriana*) co-utilized the seed eating and caching strategies of the local rodent community in the Qinling Mountains in Central China. The seeds of *Q. aliena* and *C. engleriana* were more likely to be eaten after being removed or cached than they were in situ. In contrast, *Q. glandulifera* seeds were often eaten in situ and were less likely to be eaten after being removed or cached.

Throughout our experiment, the removal and caching patterns observed at seed-release points (including small tooth marks, shallow burial, and short-distance transport) were consistent with rodent scatter-hoarding behavior and showed no evidence of interference by larger mammals. We therefore attribute the seed-removal and dispersal events recorded in this study primarily to the local rodent community.

Generally, rodents transport seeds across a variety of microhabitats, where more visited microhabitats tend to receive a larger proportion of small sized, longer-retained seeds [54]. In our study, rodents performed the processes of scatter-hoarding the seeds of each *Lithocarpus* species in distinctly different ways. We found that *L. pachyphyllus* seeds were more likely to be removed and dispersed farther than those of *L. xylocarpus* or *L. hancei*, indicating that *L. pachyphyllus* seeds received a better seed dispersal service. In counterpoint, however, these *L. pachyphyllus* seeds were more frequently removed to inferior sites under shrubs, in contrast to the seeds of *L. xylocarpus* or *L. hancei*. These patterns may relate to species-specific seed traits (e.g., seed size, tannin concentration, nutrient content, and seed-coat hardness) that influence rodent foraging decisions. Among these traits, only seed size (seed mass) was quantified in our study; tannin concentration, nutrient content and seed-coat hardness were not measured. However, the exact drivers require further investigation. Tree species produce seed with different levels of shade tolerance [55], however, shaded habitats are generally less conducive to seed germination and seedling survival [56]. This was likely a limiting factor in our study forest site, characterized by minimal canopy openness. Rodents are also more active in the understory, and thus the likelihood of seeds being eaten is greater under shrub cover [57]. Ultimately, the removal of *L. pachyphyllus* seeds to shrubs offsets the superior seed dispersal service it receives. This compensates for the overall lower removal rate of *L. xylocarpus* or *L. hancei* in relation to that of *L. pachyphyllus*, resulting in similar SDE across all three *Lithocarpus* spp. Notably, although we documented clear inter-specific differences in microhabitat destinations for the removed seeds, the number of successfully cached seeds within each microhabitat was limited by small sample sizes. For this reason, formal statistical comparisons of cached-seed proportions across microhabitat types could not be performed in the present analysis. Our seed-tracking approach records the final seed fates but cannot attribute dispersal events to individual rodent species within the local assemblage. One plausible scenario is that *L. pachyphyllus*, which bears medium-mass seeds among the three focal species, may be preferentially handled by particular rodent taxa whose home ranges are concentrated in shrub-rich microhabitats, whereas other rodent species may dominate dispersal for the remaining two *Lithocarpus* species. Different rodent taxa may diverge greatly in body size, foraging preferences, and cache-pilfering behavior, which could shape seed fate outcomes. Therefore, we treat the whole rodent community as a functional “black box” in this study. Further work combining camera trapping or individual animal monitoring would be required to disentangle species-specific plant–rodent interactions in this subtropical forest.

Seed nutritional composition and defensive secondary metabolites such as tannins are well-known drivers of rodent seed-selection decisions. However, seed nutrient and tannin concentrations were not quantified in our present experiment. Interspecific differences in seed chemical traits among these three *Lithocarpus* species constitute a plausible driver of the divergent dispersal patterns we observed, and this hypothesis needs to be directly tested in future investigations.

The equilibration of SDE among competing plant species with similar niches plays a pivotal role in facilitating species coexistence [58], especially in forest ecosystems where numerous tree species coexist and rely on scatter-hoarding rodents for seed dispersal [59]. Mechanisms underlying plant species coexistence are intricate and encompass a multitude of ecological processes. These include not only segregating seed dispersal services but also subsequent processes, such as seed germination, seedling establishment, herbivory, and seedling growth, which can similarly exhibit trade-offs and collectively influence the coexistence of sympatric species. While our study did not track final germination and establishment, the key components of complete SDE, it demonstrates that significant differentiation and trade-offs occur during the critical dispersal phase, a necessary precursor to all subsequent recruitment stages. Our findings thus provide robust, stage-specific evidence supporting the hypothesis of dispersal-niche partitioning among congeners. Future studies that integrate germination and seedling survival data across different microsites will be essential to fully parameterize SDE and validate the long-term coexistence implications suggested here. Beyond plant-recruitment monitoring, identifying which rodent species contribute to seed dispersal for each plant species is also highly valuable for fully unravelling plant–rodent mutualistic interactions in this forest system.

As a caveat, we acknowledge that our study was carried out over one year’s masting, and inter-annual variation in seed yield and in rodent population sizes and in relative species diversity will influence plant–rodent dispersal mutualisms [53,60]. Our field sampling only covered one complete over-wintering dispersal season. Seasonal food availability, inter-annual fluctuations in seed masting output, and changes in local rodent population abundance can all modify plant–rodent mutualisms and alter dispersal patterns as well as SDE. Multi-year field monitoring will be essential to test whether the dispersal-niche-partitioning pattern reported here remains temporally stable. A previous study conducted in French Guiana [61] found that *Carapa procera* (Meliaceae) seed removal by scatter-hoarding rodents was slower, pre-removal seed predation was greater, and seed dispersal distances were shorter in seed-rich years than in seed-poor years, suggesting that dispersal effectiveness is reduced under conditions of seed abundance. Similarly, another study [62] on the scatter-hoarding of *Castanopsis fargesii* (Fagaceae) seeds by rodents in the Dujiangyan region of Sichuan Province, China concluded that high seed abundance was associated with a lower rate of seed removal, which they attributed to the predator satiation hypothesis [63]. In our trial, we deployed the same number of seeds for each *Lithocarpus* spp. This may not necessarily conform to the actual proportion of seeds these species produce naturally. However, as these species generally yield abundant seeds, this may overcome any over- or under-representation of any one species, where no sampling method is completely random or unbiased [64]. Our future studies will examine whether rodent-mediated SDE similarity for *Lithocarpus* spp. is consistent between species or years, which we would predict if this dispersal niche differentiation is evolutionarily stable. Furthermore, we will extend our approach to incorporate seed germination and seedling survival monitoring [65]. Our study only quantified differences during the seed dispersal stage. Future research should integrate seedling survival rates and population dynamic models to directly test how such dispersal disparities translate into demographic advantages for species coexistence.

## 5. Conclusions

In this subtropical evergreen broad-leaved forest, three sympatric congeneric *Lithocarpus* tree species made differential use of scatter-hoarding rodent dispersal services. Despite pronounced interspecific divergence in key dispersal components, including seed removal rates, dispersal distances, and microhabitat destinations of the transported seeds, the three species achieved comparable seed dispersal effectiveness during the early seed-dispersal stage. These empirical results support the hypothesis of dispersal-niche partitioning among ecologically similar co-occurring Fagaceae species.

Nevertheless, equivalent seed dispersal effectiveness at the dispersal phase does not guarantee equivalent population recruitment success. Our study only evaluated dispersal-stage outcomes; post-dispersal processes such as seed germination, seedling survival and long-term population stability were not assessed. Additional field evidence is therefore required to verify whether this dispersal-based niche segregation contributes to long-term species coexistence in this forest community.

Interactions between plants and scatter-hoarding rodents are sensitive to environmental perturbations. Shifts in rodent community composition have the potential to break the balanced seed dispersal effectiveness among these sympatric tree species. Understanding dispersal-niche segregation thus provides useful practical insights for forest conservation and restoration under ongoing environmental change.

## Data Availability

The raw data supporting the conclusions of this article will be made available by the authors upon request.

## Supplementary Materials

The following supporting information can be downloaded at: https://www.mdpi.com/article/10.3390/ani16193145/s1, Table S1: Statistical results from generalized linear models (GLMs) and generalized linear mixed-effects models (GLMMs) with Tukey’s HSD multiple comparisons for examining differences in various seed fates-related indexes among species, with ‘species’ as the independent variable. For all indexes except Seed Dispersal Effectiveness (SDE), a GLMM approach was used with ‘seed release point’ as a random effect. For SDE, a GLM without the random effect was applied. Significance (bold) was set at *p* < 0.05.
