# Supplementary material for: Sympatric Fagaceae Segregate Rodent Scatter-Hoarding Services for Similar Seed Dispersal Effectiveness in a Subtropical Forest in China
Source: Animals (Basel). 2026 Oct 8;16(19):3145. doi: 10.3390/ani16193145 (PMC13663360; doi:10.3390/ani16193145)
Supplement: Supplementary file 1 [file animals-16-03145-s001.zip › animals-4611373-supplementary.pdf]

animals

Supplementary Materials

Table S1

Statistical results from generalized linear models (GLMs) and generalized linear mixed-effects models (GLMMs) with Tukey's HSD multiple comparisons for examining differences in various seed fates-related indexes among species, with 'species' as the independent variable. For all indexes except Seed Dispersal Effectiveness (SDE), a GLMM approach was used with 'seed release point' as a random effect. For SDE, a GLM without the random effect was applied. Significance (bold) was set at $P < 0.05$.

Seed fates	Pairwise comparison	Estimate	SE	*z*	*P*
Seed harvest	*L. pachyphyllus* - *L. xylocarpus*	384.59	2450469	0	1
	L. hancei - *L. xylocarpus*	-29.52	1732743	0	1
	L. hancei - *L. pachyphyllus*	-414.11	1732743	0	1
Seed removal	*L. pachyphyllus* - *L. hancei*	2.4501	0.5625	4.356	<0.001
	L. xylocarpus - *L. hancei*	-0.3216	0.538	-0.598	0.821
	L. xylocarpus - *L. pachyphyllus*	-2.7716	0.5639	-4.916	<0.001
Seed caching	*L. pachyphyllus* - *L. hancei*	-0.1579	0.4437	-0.356	0.933
	L. xylocarpus - *L. hancei*	0.1748	0.4705	0.371	0.927
	L. xylocarpus - *L. pachyphyllus*	0.3327	0.4646	0.716	0.754
Dispersal distance	*L. pachyphyllus* - *L. hancei*	1.4621	0.4697	3.113	0.005
	L. xylocarpus - *L. hancei*	-0.3452	0.4904	-0.704	0.7611
	L. xylocarpus - *L. pachyphyllus*	-1.8074	0.4868	-3.713	< 0.001
Percentage of seed removed (open *vs*. shrubs)	*L. pachyphyllus* - *L. hancei*	2.4395	0.777	3.14	0.005
	L. xylocarpus - *L. hancei*	-1.0665	0.7765	-1.374	0.355
	L. xylocarpus - *L. pachyphyllus*	-3.506	0.7949	-4.411	<0.001
Seed Dispersal Efficiency (SDE) †	*L. pachyphyllus* - *L. hancei*	0.008888	0.022236	0.400	0.916
	L. xylocarpus - *L. hancei*	0.003750	0.022236	0.169	0.984
	L. xylocarpus - *L. pachyphyllus*	-0.005138	0.022236	-0.231	0.971

† For SDE, results were obtained from a generalized linear model (GLM) without the random effect. For all other indexes, results were obtained from GLMMs with 'seed release point' as a random effect.

Disclaimer/Publisher's Note: The statements, opinions and data contained in all publications are solely those of the individual author(s) and contributor(s) and not of MDPI and/or the editor(s). MDPI and/or the editor(s) disclaim responsibility for any injury to people or property resulting from any ideas, methods, instructions or products referred to in the content.
